# Improving Social Media-Based Support Groups for the Rare Disease Community: Interview Study With Patients and Parents of Children with Rare and Undiagnosed Diseases

**DOI:** 10.2196/57833

**Published:** 2024-12-30

**Authors:** Tom A Doyle, Samantha L Vershaw, Erin Conboy, Colin M E Halverson

**Affiliations:** 1 Center for Bioethics Indiana University School of Medicine Indianapolis, IN United States; 2 Department of Medical and Molecular Genetics Indiana University School of Medicine Indianapolis, IN United States

**Keywords:** social media, rare disease, support groups, pediatric rare disease, Ehlers-Danlos syndrome, collagen disease, fibrillar collagen, cutis elastica, connective tissue disorders, hyperelasticity, hypermobility of joints, inherited, genetic disorder, genetics, pediatric

## Abstract

**Background:**

The rarity that is inherent in rare disease (RD) often means that patients and parents of children with RDs feel uniquely isolated and therefore are unprepared or unsupported in their care. To overcome this isolation, many within the RD community turn to the internet, and social media groups in particular, to gather useful information about their RDs. While previous research has shown that social media support groups are helpful for those affected by RDs, it is unclear what these groups are particularly useful or helpful for patients and parents of children with RDs.

**Objective:**

This study aimed to identify what specific features of disease-related support groups (DRSGs) the RD community finds particularly useful or supportive and provide a set of recommendations to improve social media–based RD support groups based on this information.

**Methods:**

Semistructured qualitative interviews were performed with patients and parents of patients with RDs. Interview participants had to be at least 18 years of age at the time of the interview, be seen by a genetics specialist at a partner health care institution and be proficient in the English language. Social media use was not a prerequisite for participation, so interview participants ranged from extensive users of social media to those who chose to remain off all social media. All interviews were conducted by phone, recorded, and then transcribed. Interview transcripts were then coded using the 6 steps outlined by Braun and Clarke. Three researchers (TAD, SLV, and CMEH) performed initial coding. After this, the study team conducted a review of themes and all members of the team agreed upon a final analysis and presentation of data.

**Results:**

We conducted 31 interviews (mean age 40, SD 10.04 years; n=27, 87% were women; n=30, 97% were non-Hispanic White). Thematic analysis revealed that social media DRSG users identified the informational usefulness of these groups as being related to the gathering and sharing of specific information about an RD, clarification about the importance and meaning of certain symptoms, and obtaining insight into an RD’s progression and prognosis. Participants also identified that DRSGs were useful sources of practical information, such as tips and tricks about managing RD-related issues and concerns. In addition, participants found DRSGs to be a useful space for sharing their disease-related stories but also highlighted a feeling of exhaustion from overexposure and overuse of DRSGs.

**Conclusions:**

This study identifies the usefulness of DRSGs for the RD community and provides a set of recommendations to improve future instances of DRSGs. These recommendations can be used to create DRSGs that are less prone to splintering into other DRSGs, thus minimizing the risk of having important RD-related information unhelpfully dispersed amongst a multitude of support groups.

## Introduction

The National Institutes of Health estimates that 25-30 million Americans, roughly 1 in 10, are affected by rare diseases (RDs). Therefore, while a single RD may impact only a small percentage of people, the culminative sum of all RDs impacts a sizable portion of the overall population. Despite this, patients with RDs often face significant barriers to appropriate care. The rarity of their conditions means that substantive information about their health is often lacking, and patients encounter significant challenges in finding and understanding the little information that is available [1]. Coupled with a paucity of appropriate diagnostic services, they face exceptionally long and difficult diagnostic odysseys [2]. This means that patients with RD find themselves on “the journey of experiencing unexplained symptoms, seeking evaluation, experiencing symptom evolution, and seeking further evaluation, all in an attempt to obtain an accurate diagnosis” [3]. Another consequence of the rarity of these conditions is that opportunities for socializing and networking between patients are few and far between. Patients therefore often feel isolated, unprepared, and unsupported in managing their care [2,4,5], as though they had been left to undertake their medical journey alone.

Given these challenges, for many patients with RDs, the internet may provide the only medium through which they can connect with others who have the same or similar diseases. Previous research has shown that patients and guardians of children with RD often use the internet, and social media in particular, as a source of informational, emotional, and social support [5-9]. Platforms that allow users to create disease-specific groups, such as Facebook (Meta) or Reddit, have been found to be especially useful for patients and parents of children with RD. Notably, a recent study found that Facebook had over 6000 user-created groups related specifically to RDs [10].

Yet, while it is evident that the members of the RD community use social media groups to engage with one another, it remains unclear what specific features of these disease-related support groups (DRSGs) make them helpful for patients and guardians of children with RD. How do patients find support within the various RD communities on social media? In order to fill this knowledge gap, we conducted a series of in-depth, qualitative interviews with patients and their guardians who were being seen in the clinic for rare genetic disorders. The results of our study are of significance for RD advocates and organizations that hope to design, establish, and cultivate social media–based RD communities.

## Methods

### Participants and Procedures

The Indiana University institutional review board approved this qualitative study. The study team consisted of three researchers: (1) one male, faculty investigator (TAD) with experience in philosophy of medicine and bioethics and doctoral-level training in qualitative research methods; (2) one male, with a doctorate in medical anthropology, advanced training in bioethics (CMEH), and extensive experience in qualitative research methods; and (3) one female research assistant (SLV) whose work focuses on improving care for the RD population.

Involvement in the study required participants to be older than 18 years of age and to have proficiency in the English language. The candidate needed either to be actively followed at Indiana University Health or to be the guardian of a minor who was being followed at Indiana University Health. Candidates were identified by genetics specialists using convenience sampling, and those individuals who expressed verbal interest in participation were scheduled for a 1-time phone interview. The recruitment strategy was agnostic as to whether candidates were intensive or casual users of social media, and even individuals who preferred not to use DRSGs at all were invited to participate. This design was meant to capture the perspectives of both individuals who do and those who do not find value in engaging in social media–based RD communities. Participants provided verbal informed consent before participating in this study. Interviews ran from April to September 2023, at which point team members agreed that the sufficient depth and richness of the data had been achieved to generate meaningful insights and themes, as is appropriate for interpretive qualitative research [11,12].

### Instruments and Analysis

The interviews were structured to learn about participants’ social media practices and values in relation to their RD needs and interests. The team jointly developed the interview guide based on a thorough literature review. The interview template consisted of approximately 21 questions outlining general uses of social media, including account platforms and perceptions of their typical social media usage; attitudes toward interviewees’ use of social media for disease information; and how social media has influenced their social lives, both as a family and individually. It also addressed any advocacy efforts in which interviewees had participated and their interactions with peers on social media through RD groups and informational sites. In order to avoid prompting participants to discuss a particular social media site or group, these sites and groups were introduced to participants in a general, nonspecific manner (eg, “on what platforms do you have a social media account?”; “are you an active member in any social media groups?”). The interview guide concluded with basic demographic questions, including gender, race, age, religious affiliation, and highest level of education. Participants could complete the interview in whatever space they felt comfortable, while the interviewer conducted the calls from a private office in order to maintain participant confidentiality. The complete interview guide used for these interviews can be found in the Multimedia Appendix 1 of this article.

One researcher (TAD) conducted the interviews while completing detailed field notes. Each interview was audio-recorded and transcribed through MacWhisper (Sindre Sorhus) or audio transcription through Microsoft Teams, both HIPAA (Health Insurance Portability and Accountability Act)-compliant, artificial intelligence–based programs. The generated transcripts were then reviewed by a member of the research team, who compared the audio recording against the transcript in order to ensure accuracy. In cases where there was a discrepancy between the audio recording and the generated transcript, the team member who conducted the interview was consulted to clarify what was said in the interview. After this, the researchers uploaded transcripts to Dedoose (SocioCultural Research Consultants, LLC), a mixed methods analysis tool especially useful for qualitative research. It was determined that reflexive thematic analysis based on the 6 steps outlined by Braun and Clarke [11] was the best method to investigate these interviews both rigorously and yet flexibly. Members of the study team familiarized themselves with the conversations and constructed a coding tree outlined by overall platform use, uses and goals of social media, information behavior, social media’s impact of perception of RD, behavior on social media, advocacy and support, mental health, and portrayal of clinicians. The codes were further enhanced, as the study team met periodically to refine and clarify themes emerging from the data, thus ensuring they were used consistently by all team members. The study team conducted a review of themes, and all members approved the final analysis and presentation of data.

### Ethical Considerations

Study procedures were approved by the Indiana University institutional review board (protocol 18779). The study was determined to be exempt research and thus consent was obtained verbally after a member of the study team thoroughly explained to participants the study procedure, the risks and benefits of participating, and how their data will be protected. Participants were also provided with this information in a study information sheet. All data presented in this manuscript has undergone proper deidentification.

## Results

### Overview

A total of 31 participants completed an interview. The average length of an interview was 45 minutes (range 21-73 minutes). After coding the data, 3 themes were identified regarding how and why DRSGs were considered valuable to patients with RDs and families, and the themes are (1) for general information about a disease and its symptoms, (2) as a source of practical advice, and (3) for accessing and managing emotional and social support. Each of these themes is elucidated in their respective sections below.

### Participant Characteristics and Demographics

Of the 31 participants who completed an interview, 8 (26%) were parents of patients with an undiagnosed or RD, and 23 (74%) were patients with an undiagnosed or RD. Overall, differences between these 2 groups were not observed; both groups shared congruent experiences that mapped onto a common set of themes and concerns.

All participants were residents of the United States. The majority identified as female (87%, 27/31) and White (97%, 30/31). The average age of participants was 40 years. Roughly half of our participants identified as Christian. Regarding educational attainment, about half had obtained a bachelor’s degree. More information about their demographics is found in Table 1.

**Table 1:** Participant demographics.

Demographics variables			Interviews (N=31), n (%)
**Gender**			
	Female	27 (87)	
	Male	2 (6)	
	Nonbinary	2 (6)	
**Religion**			
	Christian	16 (52)	
	Spiritual	5 (16)	
	None	6 (19)	
	Other	4 (13)	
**Education**			
	High school	5 (16)	
	Some college	4 (13)	
	Bachelor’s degree	14 (45)	
	Graduate degree	8 (26)	
**Race or Ethnicity**			
	Non-Hispanic White	30 (97)	
	More than one race	1 (3)	
**Age (years)**			
	Average	40	
	Range	19-63	

### Gathering Information About the Disease Process

**Figure 1:**
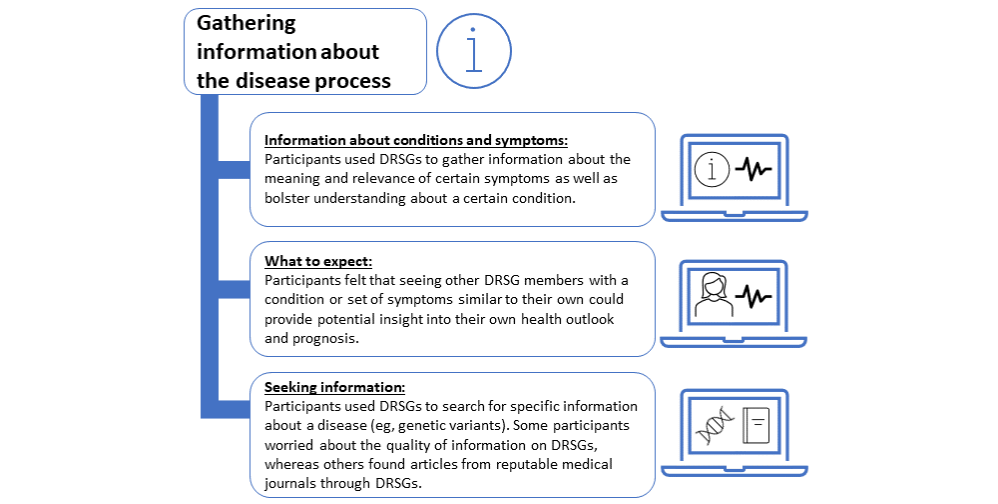
Gathering information about the disease process visual summary. DRSG: disease-related support group.

Participants often used DRSGs to gather information about their disease. In particular, they used DRSGs to gather more information about conditions and symptoms, a disease prognosis, or to seek further information about a disease (Figure 1).

### Information About Conditions and Symptoms

Participants used DRSGs to gather information about the relevance or meaning of certain symptoms as well as general information about their medical conditions. One participant remarked that social media was “a good place to start your search” [P27], and another participant stated that it could be used for “crowdsourcing medicine” [P23] insofar as one could leverage the insights offered by members of a particular DRSG in pursuit of effective diagnosis and care.

Participants recounted how joining groups for a particular condition greatly bolstered their understanding of that condition. For instance, one parent recalled the usefulness of a DRSG for gathering information about her daughter’s disorder: “It was super helpful because for us, it was kind of like, ‘Here you go, this is it.’” The group members, she said, “were pretty knowledgeable of the condition and how to manage it and work with the symptoms” [P06]. In another instance, a parent remarked that “we found out that [our daughter] had hypotonia and we didn’t know what that was, [so] I was like, ‘I’m going to look that up’, and then I saw on Facebook they have a hypotonia support group, so I did join that” [P03].

Participants also used social media groups to discover the meaning behind certain symptoms. This allowed them to relate a seemingly disparate cluster of symptoms to a particular rare condition. As one of our participants put it, “we don’t even know that our symptoms are symptoms, so you can be like, ‘Oh, yeah, that’s not normal.’ It’s validating” [P29]. In one instance, a parent of a child with an ultra-RD credited her DRSG use with linking her child’s symptoms to a specific diagnosis: “A lady […] was telling her child’s story, and I was half-listening. But then she started talking about the [symptom] and I was like, ‘What did she just say?’ and I rewound it, and then everything she said about her kid, and even a video she had of him crawling away, looked like my son” [P07]. In another case, a woman explained how she was able to use a social media group to trace “a really, really bizarre sensation” in her chest to costochondritis, a condition that is commonly experienced by those with her particular RD but one about which she had never previously heard [P12].

### What to Expect

Through their DRSG usage, our participants sought information about what they should expect when living with a certain RD or caring for a child with such a condition. One participant remarked that members of DRSGs often asked whether it was normal for their child not to reach certain developmental milestones [P01], to which other members reportedly responded by describing their own personal experiences with the condition. When asked what participants would want to get out of a DRSG, multiple interviewees cited information regarding how their particular RD progresses: “It doesn’t feel like a stagnant disease,” one participant explained, describing the unexpectedness of her child’s evolving symptoms, so she turned to social media “to just kind of compare stories” [P02]. Another participant explained that “seeing other kids, maybe that are older than [her daughter], how they’re doing and maybe the outlook on that” had been beneficial for her [P03]. Some participants, however, cautioned that hearing too much about more severe prognoses can be harmful: “You have to be in the right mindset, because you can come across very scary stories about people” [P29].

### Seeking Information

In general, our participants recalled using DRSGs to seek information about their or their child’s condition. In particular, some searched social media sites in an attempt to learn about rare genetic variants: “I search every once in a while, [for] that specific gene and tag to see if anyone else pops up” [P02]. Another participant recounted that “I did post or search for [genetic variant] and see what posts came up with that” [P01].

In contrast, participants with either medical training or a graduate degree tended to conduct research without the aid of DRSGs: “I would just go to Google Scholar. When I was at the university and I had access to the library, I would use the library to look at different articles. I might still see about access though PubMed” [P08]. Another participant, who had a background in nursing, recommended to her daughter after her diagnosis “that she had to look at more medical journal type things” rather than rely on what she could find on social media [P09]. This reticence was due to an expressed skepticism on the part of many regarding the quality of information available on such platforms. Some groups are organized for the sharing of peer-reviewed research articles, which for medically literate patients was seen as very beneficial and a way to avoid dubious information: “I can’t tell you how much I’ve learned through the [DRSG],” one young woman explained [P15].

### Gathering Practical Advice

**Figure 2:**
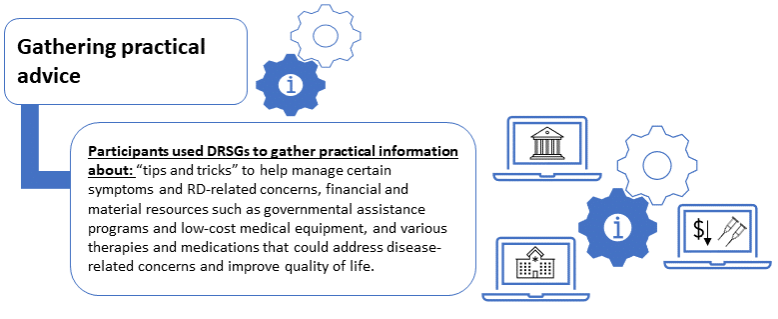
Gathering practical advice visual summary. RD: rare disease.

Participants used DSRGs to gather practical advice relating to their RD and related concerns. In particular, participants used DRSGs to gather various “tips and tricks” that were useful in addressing their disease-related concerns (Figure 2).

Participants often praised DRSGs as sources for useful “tips and tricks” to help manage disease-related issues. One participant explained that she used social media to gather practical rather than medical information: “It’s more about the giving and sharing of advice and tips” [P12]. This advice often took the form of recommendations for symptom management based on personal experience. Other group members regularly provide testimonials and appraisals of resources for managing symptoms, including everything from braces and topical creams to supportive mattresses and pillows. One parent of a child requiring a feeding tube mentioned that joining a DRSG was helpful initially after diagnosis because it allowed her to collect “a bunch of different tricks, or different clothing” [P04]. One parent of a child with ankle braces remarked: “I put on [the DRSG], ‘who would have thought that buying a pair of shoes for a two-year-old would be so difficult?’ and there was a couple of other moms who were like ‘Oh, go with this brand’” [P03]. A challenging task with the potential for significant trial-and-error related cost and frustration was averted by accessing others’ lived experiences through social media.

In addition to advice, our participants used DRSGs to gather practical resources to address disease-specific concerns. Participants mentioned DRSGs that focused narrowly on enabling users to acquire medical equipment and other materials. For instance, one interviewee described a group in which “families who have extra of different things” provided valuable supplies to families with limited financial means and access [P10]. One parent mentioned a local group whose sole purpose was to allow parents to provide others with “used equipment, or things that their kids have outgrown, or that they don’t need anymore […] and you don’t know what to do with them […] You post them on there so local people can either buy it from you or get it from you” [P07]. In other cases, resource sharing occurred through posts within general DRSGs: “People post on there if they have Aspen collars or things that they don’t need anymore,” noted one participant of a group broadly dedicated to her particular RD [P288].

Information about programs designed to assist those with disabilities was also commonly shared on DRSGs. One participant recounted that she “learned about the Medicaid waiver” for home and community-based services through DRSGs, adding that “I’m so thankful [my daughter] has that now” [P02]. This participant was also able to gather information about a program offering reduced admission to local museums for children with disabilities, and she even obtained a medical stroller and car seat through a program she learned about through a DRSG.

A large portion of our participants (74%, 23/31) reported using DRSGs to obtain or provide disease-related guidance. While the type of guidance they sought varied, participants used this information in a practical manner, either to discover an actionable therapy or to judge the usefulness of a particular medication. Some of our participants used DRSGs to collect information about therapies of which they had previously been unaware: “I hadn’t known about, for example, horseback riding therapy. I didn’t know that was a thing” [P01].

Our participants also found value in learning about others’ experiences with clinicians. One participant described the use of DRSGs to ascertain “people’s opinions about a doctor at a hospital or within a group: ‘Who has had this doctor? What is your feedback’ or ‘Have you tried this medication for [something]?’” [P02]. Such advice was seen as particularly useful, as interviewees felt that many clinicians whom they encountered were not sufficiently knowledgeable or otherwise disposed to care for their particular condition.

### Social Behavior in Social Media–Based Communities

**Figure 3:**
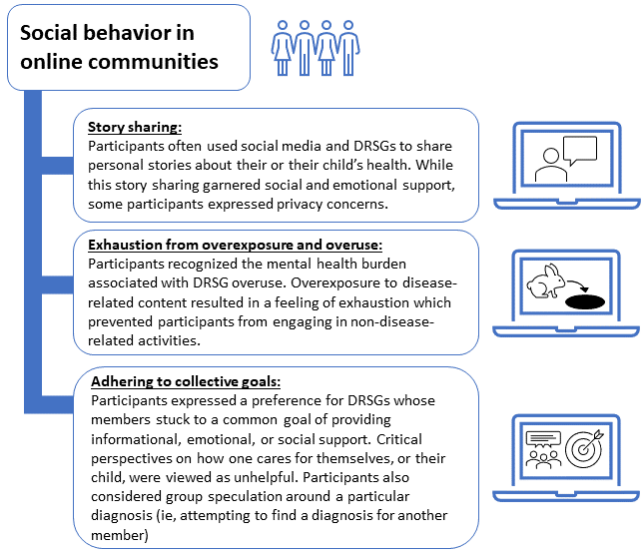
Social behavior in social media–based communities visual summary. DRSG: disease-related support group.

Participants used DRSGs to engage in social behaviors. In particular, participants used DRSGs to share stories regarding their RD experiences. However, participants also expressed concerns regarding the potential for overuse of DRSGs. Participants also believed that behavior on DRSGs should adhere to the collective goals of providing emotional, informational, and social support (Figure 3).

### Story Sharing

About half of our participants (54%, 17/31) reported that they have used social media to tell their personal stories or provide updates about their own or their child’s health. Motivations for sharing personal stories and information about health status varied. One woman used Facebook to describe her experiences in a series of posts that she had intended “for educational purposes, for awareness purposes” [P25]. A mother cited social media as “a way to keep everybody in the loop” about her child’s health, which saved her the trouble of “texting 18,000 people” to provide health updates. As another participant put it, “It’s just so much easier to put out updates like that online than it is to try to explain it 30 different times to 30 different people” [P02]. However, regardless of the participants’ motivations for sharing their personal stories, they expressed that they had received sympathetic and reassuring responses to those posts. For instance, one mother remarked, “[My daughter] had eye muscle surgery, and I did a little post, and people were saying ‘thoughts and prayers’ for her and a quick recovery” [P03].

Contrarily, some participants hesitated to share their stories so openly. “I’m very careful about what I post because I just don’t want people seeing everything. I’m private,” one participant stated [P15]. “Oversharing could be very dangerous to their own privacy as well as their own health,” noted another woman [P23].

### Exhaustion from Overexposure and Overuse

Our participants appraised social media as a generally supportive environment. However, many remarked that DRSGs can become emotionally burdensome when members spend too much time on them. They often mentioned limiting their consumption of DRSG content for that reason. One woman, for instance, stated that “there was a group of moms from the [DRSG] where we had a private group chat that would occur daily, all the time. I had to mute it. It was too much” [P06]. Another woman limited her participation in social media groups because “if I spend all my time in a chat room, that’s where my energy is going, and I had other things I wanted to do” [P24]. In fact, after leaving social media, she returned to school and graduated shortly after our interview. One parent highlighted how her DRSG use led to maladaptive and obsessive behavior. Particularly, she felt compelled to follow every lead related to her child’s condition: “It gets me looking into things to see if maybe I can find that, and I’m just like, ‘Oh, maybe I can bring this to the doctor,’ you know? And sometimes it just really takes over” [P10].

### Adhering to Collective Goals

Our participants often asserted that DRSGs have the best cohesion when members keep to the specific goal of providing informational, emotional, or social support to other members. One of our participants stated, “We’re all moms that are trying to figure out what the heck is going on with our kids. We don’t need anybody beating us up over a decision that we made or didn’t make […] Just make sure [the group is] really monitored and positive. And remember that we’re all in situations together” [P04]. Another participant expressed frustration at other members of these groups who tried to play the role of a clinician rather than providing social support or practical advice: “You have a lot of parents in these groups that I guess are pushing what they feel your child’s diagnosis is, and let’s face it: they’re not the doctor. So, I can give my information on my child, and they can be like, ‘Oh, that sounds like this.’ And then they stick to that point of they strongly feel that this is what is wrong with your child.” [P10].

## Discussion

### Principal Findings

In many cases, DRSGs provided our participants with an understanding of their RD that was informed by others’ lived experiences. This allowed them to gather practical advice regarding how others had dealt with particular symptoms, obtain general information about a disease, and socialize with those who share similar experiences. These findings support previous research on DRSGs, which has shown that online groups provide both informational and emotional support for those who share a similar diagnosis [13]. In addition, past studies have found that DRSGs provide users a space to ask and answer specific questions about medication management, hospitalizations, and clinic visits as well as provide a supportive environment where users can disclose personal stories about their disease experience [14].

Due to random sampling used during the recruitment process, our study was also able to capture the perspectives of those who do not regularly use social media. This has been an oversight in much previous research [5-7,15,16]. We found that these participants avoided social media either due to privacy concerns or a perceived lack of benefit. Privacy concerns have been found in other studies, though they do not always rise to the level of stopping patients from participating in DRSGs [5,15,16].

### Information About the Disease Process

Within the existing literature on RD and social media use, it has been found that users often turn to DRSGs for information about a diagnosis or test results or to participate in conversations about a specific diagnosis [6,7]. Another study reported that patients with RDs use DRSGs primarily as a source to share medical information about symptoms, treatments, and diagnoses [8].

Our study likewise found that participants regularly made use of social media for informational needs. However, they did not expect this information to replace communication with trusted medical professionals. Many participants understood the role of the community in these groups not as that of experts, but rather as individuals with unique and personal insight into what it is like to live with and care for someone with an RD. This aligns with previous research which has found that participants often regarded clinical advice accessed through social media as dubious and to be met with skepticism [5].

DRSGs, then, were seen not as a replacement for recommendations offered by a participant’s clinician, but rather as a complement to these clinical recommendations. This finding is congruent with a review of previous research which has found that patients often used social media–based communities to receive information and advice on how to improve their medical care or explore treatment options [17]. One study found that patients often turned to online support groups because they believed their providers lacked a sufficient amount of time to discuss each treatment option in depth. These groups allowed patients to gather information from members of the community who were actively undergoing these treatments [18]. Our participants engaged in similar behavior, which indicates that the patient perspectives gathered from DRSGs parallel information discussed in a clinical setting.

Many participants preferred internet-based databases sources such as Google Scholar or PubMed as a source of information rather than social media, due to the credibility they attached to them. Information from medical journals was generally felt to be more reliable and trustworthy than information found through the self-report of DRSG members. Such a finding corroborates a recent review of the literature regarding online health information–seeking behaviors which highlighted the central role of trust in determining patients’ information-seeking behavior [19].

One critical finding from our study is that information-seeking through social media was often seen as burdensome and could even become maladaptive. Our participants reported using the search feature on DRSGs to find information about particular genetic findings, but they also highlighted that such searching could also become obsessive and result in mental exhaustion.

### Practical Advice and Lived Experience

While it is evident that our participants engaged in information sharing and gathering, it is important to emphasize that our participants often used DRSGs as more than just an informational resource. In particular, they relayed these groups’ usefulness for obtaining practical advice, for providing and seeking appraisal of medical providers, as well as for sharing and gathering guidance on how to enroll in certain government programs or to obtain certain government benefits. These “tips and tricks” allowed them to navigate and address their many concerns that fall outside of the scope of traditional clinical care. They additionally provide information related to specific products that particular RD communities have found useful, such as brands of clothing or specific kinds of medical braces. Moreover, participants sought information about others’ lived experiences in order to forecast what to expect regarding disease progression, assisting in personal prognostication.

While previous research has identified RD-specific DRSGs as a useful resource for such general practical advice [5], one of our study’s novel findings is that DRSGs are important sources of material exchange. While much of the literature focuses on social media as a place to obtain information and social support, our participants relayed the importance of DRSGs in the selling, buying, and trading of specific medical equipment. Similar to this, an additional novel finding of our study was the use of DRSGs to discover and help obtain various material and service benefits offered by federal and state governments. Previous cost-of-illness research has established RDs as a major economic burden for patients and their families [20-22]. Hence, it is noteworthy that DRSGs have the potential to alleviate particular disease-related economic burdens. Conceivably, DRSGs allow users to recuperate some of the costs associated with medical equipment by selling them directly to other patients and their guardians; in turn, those individuals may do so at a reduced price and thus do not incur the often-exorbitant cost of new equipment.

### Socializing and Networking

As made evident by our study, social media serves as one possible venue for patients and their guardians to establish and maintain social connections despite physical, geographic, and medical barriers. Our findings concur with these previous findings that social media sites that allow users to create disease-specific support groups, such as Facebook or Reddit, can be helpful resources for those with chronic diseases [23,24]. Social support has been shown to have a positive impact on one’s health [25]. In particular, social support can reduce psychological distress, mitigate feelings of loneliness, improve quality of life, and decrease mortality risk [26]. In light of this fact, many health organizations, such as the World Health Organization (WHO) and the US Department of Health and Human Services (DHHS), now emphasize the importance of a person’s ability to establish and maintain social relationships with members of their community [27,28]. For this reason, the WHO has recently established a Commission on Social Connection in order to address social isolation and loneliness and the DHHS has established the objective of increasing social and community support as part of its Healthy People 2030 longitudinal-objective program [29,30]. DRSGs therefore have the ability to play a critical role in improving the overall health and connectedness of RD communities.

Another novel finding of our study was participants’ preference for DRSGs that adhere to the perceived objectives or goals of a support group. That is, many of our participants understood the overall purpose of these groups as providing a supportive and non-judgmental space for patients and caregivers to engage in discussions about a particular disease and its symptoms, practical tips and tricks that could improve the overall quality of life, and an environment in which they could share their personal experience with a disease if they so choose. Our participants found it off-putting when discussions deviated from these goals.

### Recommendations

Quantitative analysis has shown that thousands of RD social media groups now exist [10]. While this staggering number may provide a diverse range of opportunities for informational, emotional, and social support, it also suggests that valuable resources are unhelpfully distributed across an unmanageable number of virtual locations. For instance, the aforementioned quantitative analysis found 93 unique support groups specifically for a single rare connective tissue disorder, and that number accounts only for groups dedicated to its pediatric form [10]. This suggests that patients are faced with the burden of having to figure out which specific support groups to join, how to resolve possible conflicts in information gathered from these various groups, and how to avoid oversaturating their social media feeds with disease-related information from disparate groups, which we have found is often emotionally burdensome.

It is evident, then, that the duplication of efforts is a serious problem facing DRSG administrators and their users. We contend that a solution to this substantial problem is to ensure that DRSGs are designed and operated in a manner that ensures users’ informational and social needs are fulfilled by a single group. It is likely that they will then be less likely to create new, schismogenic DRSGs, thus alleviating the problem of duplicating efforts and stretching virtual resources too thin.

Based on the data from our interviews, we propose the following recommendations for creating a DRSG that patients with RDs and their guardians will find valuable, usable, and worthwhile (Table 2). While the scope of our study was limited to RD, it is possible that these recommendations may be generalizable to other, non-RD social media–based communities.

**Table 2:** Recommendations to Improve DRSGs^a^.

DRSG usability issue	Potential solution	Explanation	Usefulness
Our results show that DRSGs are commonly used to gather practical or general advice related to a particular rare disease. This Important information may become “buried” by numerous new posts which may lack such practical relevance.	Establish and make use of pinned posts.	Posts that contain information, practical advice, or resources that the community finds particularly helpful should be “pinned” at the top of the group page.	Previous research has shown pinned posts to be a useful way to highlight and propagate important information [31]. Pinned posts can prevent members from repeatedly asking the same important questions. This allows new members to find the most helpful and important information as soon as they become a part of the group.
Certain materials, such as applications for governmental benefits, information about disability assistance programs, and other similar documents may not get proper attention when simply shared through a single post. DRSG users have identified these documents and resources as being particularly helpful to them.	Establish and make use of a “Files” section.	Official resources and documents should be archived in a set of group files. Group files can either be integrated through the social platform itself (eg, through the “Files” feature on Facebook) or through a third-party document hosting service (eg, Google or Dropbox).	A “Files” section allows users to share and store documents related to various assistance programs. This ensures that users can easily find and access important documents or resources. In addition, a “Files” section can allow DRSG users to share articles from journals for members of the community who possess a high health literacy.
In order to find relevant information related to a specific medication, therapy, symptom, or medical concern, DRSG users are often forced to conduct an imprecise search of all posts in a particular group and decipher them for potential relevance.	Introduce and encourage the use of posttags.	To enhance the searchability of the group on a particular topic, users should be encouraged to put particular keywords at the beginning of their posts that summarize the overall theme or topic of the post.	Prior research [32] has highlighted the usefulness of tags in enhancing searchability [33,34]. Posttags can assist users in searching quickly for a particular symptom, medication, or disease-related concern. This prevents repeated posting about similar issues or symptoms.
Our participants described the overuse of DRSGs as leading to exhaustion and emotional burden.	Creation and amplification of a community guide.	A community guide directs users on how to navigate the DRSG and how to make use of its various features and materials. This guide can also indicate the dangers of overuse (ie, mental exhaustion) of social media.	A community guide can help to ensure that users are “getting the most” out of a DRSG while ensuring that they do not feel overwhelmed by an abundance of information. In addition, including a section on the importance of taking breaks from social media can help users maintain healthy DRSG use.
DRSG users expressed frustration when other group members did not adhere to the perceived goal of the group (eg, to provide a supportive environment for patients and caregivers).	Creation of a group mission statement and a clear explanation of moderation decisions based on that mission statement.	In conjunction with a set of standard rules to maintain proper group decorum, a mission statement establishes the specific goals of the group and allows moderators to ensure the community focuses on these goals.	Establishing a mission statement for a DRSG can ensure that users are mindful of the specific goals and objectives of a group, preventing them from sharing irrelevant or disruptive content in an otherwise supportive environment.

^a^DRSG: disease-related support group.

Aside from these more targeted improvements for DRSGs, clinicians and RD advocates should also be aware of the importance of patient autonomy and community sovereignty when creating or recommending support groups. While our participants expressed a preference for competent moderation and proper group governance, it was also evident that they appreciated the ability to behave in an authentic or organic manner within these groups. While clinicians and advocacy organizations have a role to play in patient-oriented social media, it is important to recognize that this role should not impede a community’s or patient’s ability to express concerns or criticisms about specific clinicians or advocacy organizations in these virtual spaces. Therefore, clinicians and advocacy organizations that either create or engage in DRSGs must recognize that a social media–based support group truly belongs to a community of patients rather than to their organizational or clinical agenda. By asserting that a group belongs to the community and its members, clinicians and advocates help to empower patients and caregivers [35,36].

Therefore, we recommend that clinicians and advocacy organizations work to develop plans to ensure that DRSGs foster patient autonomy and community sovereignty rather than diminish it. With respect to group governance, this means giving community members a say in group moderation. Kiesler and colleagues offer useful guidance on this point [37]: They provide a total of 33 “design claims” that offer insight into the effective regulation of online spaces. Of particular relevance, they highlight that moderation decisions from members of the community are viewed as more legitimate and more effective, and that rule making which involves and incorporates the community’s feedback allows for better compliance with rules.

### Conclusion

Our study provides insight into the specific ways that patients and parents of children with a RD interact with DRSGs. We found that DRSGs were used to collect and seek medical information about a disease and its symptoms, gather practical resources and advice, and socialize and network with those who share a similar experience to their own regarding a particular disease. Importantly, these results uncover and highlight the particular features and elements of DRSGs that members of the RD community find helpful, usable, and worthwhile. Through considering the practical importance of these results, we established a set of recommendations that are tailored specifically to DRSGs. These recommendations provide guidance to RD advocates and organizations that manage DRSGs, which may reduce the reduplication of efforts and the splintering of disease-related support across an unmanageable multitude of groups, which at present is often experienced as time-consuming and emotionally burdensome.

### Limitations

Our sample population featured a significant gender and race skew, with participants predominantly identifying as female and White. The collected data may therefore not necessarily represent the diverse perspectives present in the RD community. This skew could also impact the generalizability of our results to the many DRSGs found across various social media platforms. In addition, limitations related to scope and interview length prevented our study from examining how participants discovered support groups and whether certain search strategies or terms yielded information about potentially relevant social media–based support groups.

## Appendix

Multimedia Appendix 1 Interview Questions.

## Abbreviations

DHHS: Department of Health and Human Services
DRSG: disease-related support group
HIPAA: Health Insurance Portability and Accountability Act
RD: rare disease
WHO: World Health Organization

## Data Availability

The datasets generated during and/or analyzed during this study are not publicly available due to the private and sensitive nature contained in the qualitative data. Relevant sections of deidentified data and/or coding trees are available from the corresponding author upon reasonable request.
