# Supplementary material for: Improving Social Media-Based Support Groups for the Rare Disease Community: Interview Study With Patients and Parents of Children with Rare and Undiagnosed Diseases
Source: JMIR Hum Factors. 2024 Dec 30;11:e57833. doi: 10.2196/57833 (PMC11730222; doi:10.2196/57833)
Supplement: Multimedia Appendix 1 [file humanfactors_v11i1e57833_app1.docx]

**SOCIAL MEDIA: GENERAL USE**

1. On which platforms do you have an active social media account?
   1. **How long have you had each of those accounts?**
   2. **Tell me about how you use each of these accounts. What role do they play in your life?**
   3. **Has your use of any of those accounts changed over time? If yes: why?**
   4. **Tell me about how you feel about each of these accounts. How does using them make you feel?**
2. On what device(s) do you view your social media accounts?
3. What content do you consume on your social media accounts?
   1. PROMPT: disease-related? Humor? News? Etc.
4. **Are you an active member in any social media groups?**
   1. **If yes, what groups are you an active member in?**
   2. **Do you feel like you primarily post or consume/lurk in the social media groups of which you are a member?**
5. Do you prefer to communicate online through comment sections, direct messaging (incl. email), posts in private groups, public posts, or all/multiple of the above? (Or none, or other)
6. Do you notice any trends in what types of posts you engage with most (like, comment, share…) on social media?
7. How do you feel about how much time you spend on social media per day?
   1. Do you feel like it is too much or too little?
   2. Have you ever tried to limit your time on social media? And if so: why?

**SOCIAL MEDIA AND MEDICAL CONDITION**

1. Do you feel that social media has improved, made worse, or left the same your perception or attitude toward your/your child’s symptoms or condition in any way?
2. Have your/your child’s symptoms affected your in-person social life?
   1. Have they affected your social life online?
3. Have you used social media to provide others with more information about your/your child’s condition or to tell your story?
4. Have you used social media for research or to gather more information about your/your child’s condition or about your/your child’s healthcare?
   1. **Have you used information gained on social media to help change your/your child’s care?**
      1. If yes: Where did you gather this information?
      2. If yes: Was it useful to you?
5. Have you ever encountered information related to healthcare on social media that made you skeptical?
   1. Where did you find this information?
   2. How did you proceed? Did you do more research? Did you talk to a knowledgeable person about it?
   3. Did you say anything about it to anyone else?

**SOCIAL LIFE AND ADVOCACY**

1. Have you used social media for support or advocacy?
2. Have you ever tried to find an advocacy group but were unable?
3. Have you used social media to improve your social life?
   1. PROMPT: In what ways? (friends, content, resources…)

**BEHAVIOR ONLINE**

1. How, if at all, do you feel like people behavior differently online versus in person?
2. How, if at all, do you feel like *you* specifically behave differently online versus in person?
   1. Do you feel like you have different kinds of social encounters online than in person?
   2. Have you ever encountered a “troll” online, related to rare disease?
3. Have you ever had a negative encounter online?
   1. IF YES: Tell me about it.
   2. IF YES: Do you feel this encounter transpired differently from how it would have happened in person?
   3. Do you have strategies for dealing with negative experiences online?
   4. Do they differ from your strategies for dealing with negative, in-person experiences?
4. In online rare disease groups, have you encountered differences in opinion about who can be a member of these groups?
   1. How have those differences been addressed?
   2. Do you feel that there is gatekeeping on any rare disease groups on social media?
   3. Do you feel gatekeeping is positive, necessary, or negative?
5. In online rare disease groups, have you encountered differences in opinion about the goals of the group?
   1. How have those differences been addressed?
6. Have you ever felt bad after logging off of a social media site or closing a social media app?
   1. If so, what led up to that?

**DEMOGRAPHICS (if unknown)**

1. How would you describe your gender?
2. Do you consider yourself religious or spiritual?
   1. If so: Do you identify with a particular denomination or group?
3. What is your highest level of education?
4. How would you describe your race/ethnicity?
5. How old are you?
